# Inhibition of PHLPP1 ameliorates cardiac dysfunction via activation of the PI3K/Akt/mTOR signalling pathway in diabetic cardiomyopathy

**DOI:** 10.1111/jcmm.15123

**Published:** 2020-03-09

**Authors:** Mingjun Zhang, Xuyang Wang, Ming Liu, Dian Liu, Jinyu Pan, Jingjing Tian, Tao Jin, Yunfan Xu, Fengshuang An

**Affiliations:** ^1^ Department of Cardiology The Key Laboratory of Cardiovascular Remodeling and Function Research Chinese Ministry of Education Chinese National Health Commission and Chinese Academy of Medical Sciences The State and Shandong Province Joint Key Laboratory of Translational Cardiovascular Medicine Qilu Hospital of Shandong University Jinan China; ^2^ Department of Cardiology Shandong Provincial Qianfoshan Hospital of Shandong First Medical University Jinan China; ^3^ College of Life Sciences Sichuan University Chengdu China

**Keywords:** apoptosis, diabetic cardiomyopathy, fibrosis, PHLPP1, PI3K/Akt/mTOR signal

## Abstract

**Background:**

Pleckstrin homology (PH) domain leucine‐rich repeat protein phosphatase 1 (PHLPP1) is a kind of serine/threonine phosphatase, whose dysregulation is accompanied with numerous human diseases. However, its role in diabetic cardiomyopathy remains unclear. We explored the underlying function and mechanism of PHLPP1 in diabetic cardiomyopathy (DCM).

**Method:**

In vivo*,* Type 1 diabetic rats were induced by intraperitoneal injection of 60 mg/kg streptozotocin (STZ). Lentivirus‐mediated short hairpin RNA (shRNA) was used to knock down the expression of PHLPP1. In vitro, primary neonatal rat cardiomyocytes and H9C2 cells were incubated in 5.5 mmol/L glucose (normal glucose, NG) or 33.3 mmol/L glucose (high glucose, HG). PHLPP1 expression was inhibited by PHLPP1‐siRNA to probe into the function of PHLPP1 in high glucose‐induced apoptosis in H9c2 cells.

**Results:**

Diabetic rats showed up‐regulated PHLPP1 expression, left ventricular dysfunction, increased myocardial apoptosis and fibrosis. PHLPP1 inhibition alleviated cardiac dysfunction. Additionally, PHLPP1 inhibition significantly reduced HG‐induced apoptosis and restored PI3K/AKT/mTOR pathway activity in H9c2 cells. Furthermore, pretreatment with LY294002, an inhibitor of PI3K/Akt/mTOR pathway, abolished the anti‐apoptotic effect of PHLPP1 inhibition.

**Conclusion:**

Our study indicated that PHLPP1 inhibition alleviated cardiac dysfunction via activating the PI3K/Akt/mTOR signalling pathway in DCM. Therefore, PHLPP1 may be a novel therapeutic target for human DCM.

## 1 INTRODUCTION

Diabetes mellitus is one of the most important events to cause mortality and morbidity all over the world,^1^ representing a global burden on human health and economics. Over half of diabetics die from cardiovascular disease, including diabetic cardiomyopathy (DCM).^2^ DCM, unique to other complications such as hypertension as well as coronary artery disease,^3^, ^4^ is distinguished by left ventricular (LV) hypertrophy, LV systolic and diastolic dysfunction.^5^, ^6^, ^7^ The pathogenesis of diabetic cardiomyopathy is complicated, including oxidative stress,^8^, ^9^ interstitial fibrosis^10^ and myocardial apoptosis.^2^ Above all, chronic hyperglycaemia is the most prominent pathophysiological stimuli involved in the development of DCM.^11^, ^12^ However, the molecular mechanisms of chronic hyperglycaemia‐induced cardiomyocyte apoptosis remain to be elucidated.

Pleckstrin homology (PH) domain leucine‐rich repeat protein phosphatase 1 (PHLPP1), a newfound serine/threonine phosphatases, is a member of the type 2C phosphatase (PP2C) family.^13^ It is well known that PHLPP1 plays a significant role in suppressing cell survival.^14^, ^15^, ^16^, ^17^ And mounting evidence support the fact that PHLPP1 could directly dephosphorylate the hydrophobic motif of Akt (Ser473) which will lead to the inhibition of kinase activity and suppress the occurrence and progression in several cancers, such as colon cancer,^14^, ^18^ prostate cancer,^15^ pancreatic cancer^19^ and lymphoma.^20^ Moreover, a previous study stated that impaired insulin action could increase PHLPP1 level in the adipose and muscle tissues of obese patients.^21^ While many studies have studied the function of PHLPP1, the role of PHLPP1 in DCM has not been reported yet.

The phosphoinositide 3‐kinase (PI3K)/protein kinase B (Akt)/mammalian target of rapamycin (mTOR) signalling pathway is central to regulating cell transcription, metabolism, survival and inflammation.^22^, ^23^ It is well established that during diabetes, defective insulin signalling down‐regulates the activity of PI3K,^24^ leading to Akt and mTOR inactivation. Previous studies have demonstrated that Akt is closely related to PHLPP1 in cell survival and inflammation.^25^ However, the role of the PHLPP1 in PI3K/Akt/mTOR signal pathway in the process of DCM has not been investigated.

Here, we hypothesized that PHLPP1 inhibition is likely to have a protective effect in DCM. To make clear the role of PHLPP1 in the development of DCM, we investigated in primary rat cardiomyocytes as well as H9c2 cells induced by high glucose in vitro and in a DCM rat model in vivo.

## 2 MATERIALS AND METHODS

### 2.1 Animals

Sixty male 4‐week‐old Sprague Dawley (SD) rats purchased from Beijing Weitong Lihua Experimental Animal Technology (Beijing, China) were housed at 22 ± 2°C under an alternating 12‐hour light/dark cycle with free access to water and rat chow. After adaptive feeding with standard experimental rat chow for 1 week, all rats were randomly grouped into the following four (n = 15 for each group): control, diabetes mellitus (DM), DM + shRNA‐negative control (shN.C) and DM + shRNA‐PHLPP1. All diabetic rats were induced by a single intraperitoneally injection of high‐dose streptozotocin (60 mg/kg, STZ, Solarbio) dissolved in citrate buffer (0.5 mL, pH 4.5), and the control group was administered with the same volume of citrate buffer. One week after STZ administration, glucometer (Roche) was used to measure tail vein glucose levels. Only rats with blood glucose ≥16.7 mmol/L were considered as type 1 diabetic rats. Twelve weeks later, an amount of 1 × 10^8^ UT/50 μL of lentivector with PHLPP1 shRNA (GenePharma) or 50 μL lentivehicle (GenePharma) was injected into the jugular vein. Four weeks after PHLPP1 shRNA injection, rats were anaesthetized with 3% pentobarbital sodium intraperitoneally (35 mg/kg) and then killed. Hearts were weighted and immersion fixed in formalin for the following histology analysis. All experiments conformed to animal protocols approved by the Shandong University Animal Care Committee. The study protocol was approved by the Institutional Ethics Committee of Shandong University. The PHLPP1 shRNA sequence was 5′‐CUACCCAGUUCCAAAUUAUTT‐3′ (GenePharma). The negative control sequence was 5′‐TTCTCCGAACGTGTCACGT‐3′ (GenePharma).

### 2.2 Cardiac function measurement

Rats were anaesthetized by inhalation of isoflurane gas (2.5%) until immobile in the imaging laboratory and kept in anaesthesia with 1.5% isoflurane by a nose cone connected to the anaesthesia machine. Cardiac function was evaluated with the Vevo770 imaging system (VisualSonic) before lentivirus infection and before killing. The left ventricular ejection fraction (LVEF), fractional shortening (FS), peak E, peak A, early (E′), late (A′) and left ventricular end‐diastolic dimension (LVEDd) were measured. The ratio of early‐to‐late mitral flow velocity (E/A) and diastolic velocity ratio (E′/A′) were calculated.

### 2.3 Histology and immunohistochemistry

The formalin‐fixed and paraffin‐embedded rat heart tissues were cut into 5 μm sections for the following analyses. Heart sections were stained with Haematoxylin and eosin (HE) to measure cardiomyocyte width. Masson's trichrome and Sirius red staining was performed to observe interstitial collagen deposition. The cardiomyocyte cell diameter and fibrotic area were quantified by ImageJ software, and 500 cells per rat were measured for analysis. The slides were incubated overnight at 4°C with the specific primary antibodies against PHLPP1 (dilution, 1:50; Proteintech, 22789‐1‐AP), collagen I (dilution, 1:200; Abcam) and collagen III (dilution, 1:500; Abcam) for immunohistochemistry. The next day, the samples were incubated with secondary antibodies for 30 minutes at 37°C to detect the expression of PHLPP1, collagen I and collagen III.

### 2.4 TUNEL assay

Apoptotic cells in myocardium were detected with a in situ cell death detection kit (Roche) following the manufacturer's instructions. Heart sections were deparaffinized, hydrated and subsequently underwent permeabilization solution (0.1% Triton X‐100) for 5 minutes. H9c2 cells were fixed with 4% paraformaldehyde for 20 minutes at room temperature, incubated in TUNEL reaction mixture for 60 minutes at 37°C and then sealed in Prolong Gold Anti‐Fade Reagent with DAPI (Invitrogen). Images were acquired via a fluorescence microscopy (Nikon).

### 2.5 Cell culture

Primary cardiomyocytes were obtained from neonate SD rats (2‐3 days old, Shandong University). The neonatal rat ventricular tissues were digested with collagenase type II (Solarbio) dissolved in D‐hanks solution (Gibico). Then, the digested cells were placed in Dulbecco's modified Eagle's medium (DMEM, 1 g/L glucose) with 8% horse serum and 5% calf serum for 2 hours at 37°C in a humidified atmosphere containing 5% CO_2_. After the incubation, the unattached cells were collected and considered as primary cardiomyocytes, while the attached cells were considered to be myocardial fibroblasts and discarded. After the following 3‐4 days, the primary cardiomyocytes were incubated in serum‐free DMEM for 8 hours before treatment with individual procedure. H9c2 cardiomyoblasts were passaged every 2 days and seeded in 6‐well culture plates. When cell populations reached 50% confluence, the medium was substituted with FBS‐free DMEM for 4 hours serum starvation. After that, cells were incubated in normal glucose (NG; 5.5 mmol/L glucose) or high glucose (HG; 33.3 mmol/L glucose) and harvested at 6, 12, 24 and 48 hours of HG treatment. To explore the potential role of PHLPP1 on PI3K, Akt and mTOR phosphorylation as well as on cardiomyocytes apoptosis, PHLPP1‐siRNA plasmids were transfected into H9c2 cardiomyoblasts 24 hours prior to HG stimulation. Moreover, in order to explore the potential role of PHLPP1 in PI3K/Akt/mTOR pathway under HG stimulation, a specific PI3K inhibitor LY294002 (MCE, HY‐10108, 25 μmol/L) was added to the H9c2 cardiomyoblasts medium 2 hours prior to the HG (33.3 mmol/L) treatment.

### 2.6 Measurement of ROS

2′,7′‐dichlorofluorescein diacetate (DCFH‐DA, Sigma) was used to observe ROS production. Positive control group was treated with Rosup (50 mg/L) 30 minutes before observation. Experimental group was treated with NAC (3 mmol/L) 2 hours before HG stimuli. After HG groups were incubated with HG for 6 hours, H9c2 cardiomyoblasts were exposed to DCFH‐DA (10 μmol/L) for 30 minutes at 37°C. Fluorescence intensity was observed via a fluorescence microscope (Nikon).

### 2.7 Immunofluorescence microscopy

Primary neonatal rat cardiomyocytes and H9c2 cardiomyoblasts were fixed with 4% paraformaldehyde, permeabilized and then blocked in 5% goat serum. Cells were incubated with rabbit anti‐PHLPP1 (1:50 dilution; Proteintech, 22789‐1‐AP) overnight at 4°C and incubated with a secondary antibody (1:300 dilution; CST) for 30 minutes at 37°C. Finally, cells were sealed in Prolong Gold Anti‐Fade Reagent with DAPI (Invitrogen) after being washed with PBS. Images were acquired via an immunofluorescence microscopy (Nikon).

### 2.8 PHLPP1 siRNA production and infection in H9c2 cardiomyoblasts

The expression of PHLPP1 in H9c2 cardiomyoblasts were inhibited by PHLPP1‐siRNA plasmids with the help of Lipofectamine 2000. The specific oligoribonucleotides used to inhibit PHLPP1 synthesis were 5′‐CUACCCAGUUCCAAAUUAUTT‐3′ and 5′‐AUAAUUUGGAACUGGGUAGTT‐3′. The negative control was 5′‐UUCUCCGAACGUGUCACGUTT‐3′ and 5′‐ ACGUGACACGUUCGGAGAATT‐3′. (Gene Pharma).

### 2.9 Western blot analysis

Equal amounts of protein were obtained from freshly dissected rat hearts, primary neonatal cardiomyocytes and H9c2 cardiomyoblasts following the procedure of BCA Protein Assay Kit (Solarbio). And then, the protein was separated through 8% or 10% sodium dodecyl sulphate‐polyacrylamide gel electrophoresis (SDS‐PAGE) before transferred to PVDF membranes (Millipore). The membranes were blocked in 5% non‐fat milk for 90 minutes at room temperature and then incubated at 4°C with specific primary antibodies against GAPDH (Abways, P04406), PHLPP1 (Proteintech, 22789‐1‐AP), collagenI (novusbio, NBP1‐30054), collagenIII (novusbio, NB600‐594SS), MMP2 (Proteintech, 10373‐2‐AP), MMP9 (Proteintech, 10387‐2‐AP), cleaved caspase‐3, Bcl2‐associated X protein (Bax) (CST, 2772), B‐cell lymphoma/leukaemia‐2 (Bcl‐2) (Affinity, AF6139), p‐PI3K (CST, 4228), p‐Akt (abcam, ab38449), p‐mTOR (abcam, ab109268), PI3K (CST, 4292), Akt (abcam, ab179463) and mTOR (abcam, ab134903) for detection.

### 2.10 Real time RT‐PCR

Total RNA samples were extracted from rat hearts by Trizol reagent (Ambion) following the manufacturer's protocol. qRT‐PCR was performed using the Primescriopt™ RT reagent kit with gDNA Eraser (TakaRa). The PCR primes for β‐MHC were 5′‐GCCAACTATGCTGGAGCTGAT‐3′ (forward) and 5′‐TGTCCATCACCCCTGGAGAC‐3′ (reverse). The PCR prime for BNP was 5′‐TTAGGTCTCAAGACAGCGCC‐3′ (forward) and 5′‐TAAAACAACCTCAGCCCGTCA‐3′ (reverse). β‐actin gene was selected as the endogenous reference using the primes 5′‐CTCTGTGTGGATTGGTGGCT‐3′ (forward) and 5′‐CGCAGCTCAGTAACAGTCCG‐3′ (reverse). All experiments were repeated at least three times. The 2‐ΔΔCT method was used to calculate the fold changes of β‐MHC and BNP.

### 2.11 Statistical analysis

Each experiment was performed at least 3 times. Data are reported as the means ± standard deviation. Differences between two groups were performed by unpaired *t* test, and multiple groups involved one‐way ANOVA followed by Scheffe's test or Bonferroni's post hoc test or Dunnet's multiple‐to‐one comparison test. *P* < .05 was considered as statistically significant. All statistical analyses were carried out using Prism 6.0 (Graphpad) and SPSS 20.0.

## 3 RESULTS

### 3.1 Diabetes increased myocardial PHLPP1 expression and PHLPP1 down‐regulation prevented diabetes‐induced myocardial remodelling

**Figure 1:**
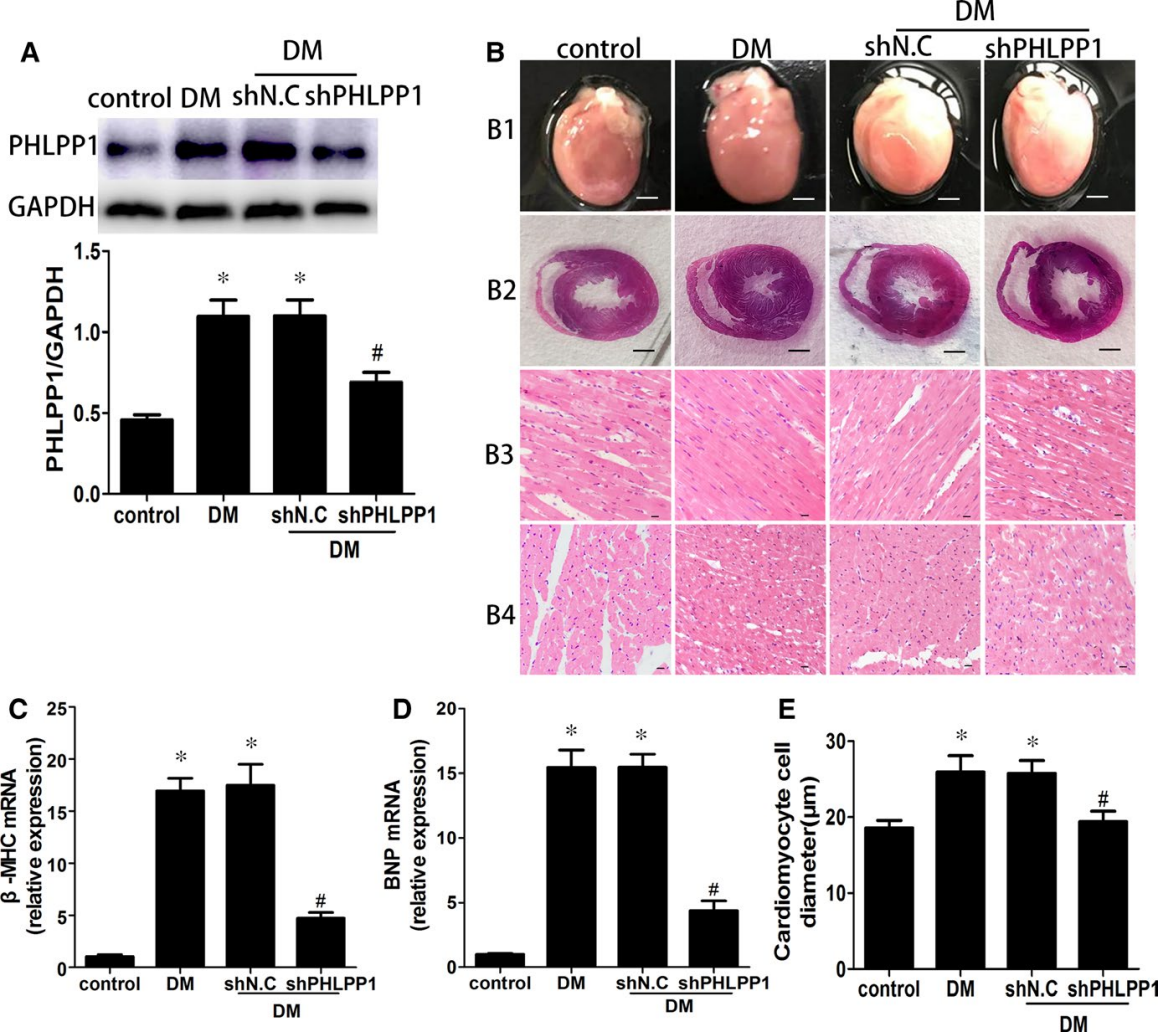
PHLPP1 expression and pathology of control and diabetic rat hearts. A, Western blot analysis of PHLPP1 protein levels (n = 6 per group). B1, Heart size (scale bar: 3 mm, n = 8 per group). B2, HE staining of cross shaft of musculi papillares in heart (n = 8 per group). B3, Representative haematoxylin and eosin staining (HE) of longitudinal left ventricular (LV) sections (scale bar: 20 μm, n = 8 per group). B4, Representative HE staining of LV transverse sections (scale bar: 20 μm, n = 8 per group). C, Relative mRNA fold changes of β‐MHC (n = 6 per group). D, Relative mRNA fold changes of BNP (n = 6 per group). E, Quantitative analysis of cardiomyocyte cell diameter (n = 8 per group). Control: normal rats. DM: diabetes mellitus. shN.C: negative control shRNA. shPHLPP1: PHLPP1 shRNA. All experiments were performed at least 3 times. Data are expressed as the means ± SD. Statistical analysis was performed using one‐way ANOVA followed by Bonferroni's post hoc test. *P < .05 compared with controls; #*P* < .05 compared with DM or shN.C in DM

PHLPP1 protein level in the diabetic rat heart was much higher than that in controls, and the PHLPP1 expression was reduced in shPHLPP1‐treated diabetic rat hearts compared with vehicle‐treated diabetic rats as demonstrated by Western blotting (*P* < .05; Figure 1A). HE stain showed that PHLPP1 down‐regulation restored the increment of cardiomyocyte cell diameter (*P* < .05; Figure 1B,E). Moreover, qRT‐PCR indicated that diabetes‐induced up‐regulation of β‐MHC and BNP was reduced after shPHLPP1 treatment. (*P* < .05; Figure 1C,D). Finally, the ratio of heart weight to bodyweight was increased in diabetic rats than controls (*P* < .05; Table 1). And the ratio of heart weight to bodyweight of shPHLPP1‐treated diabetic rats appeared to be lower than that of the untreated diabetic rats, but this difference did not achieve statistical significance (Table 1).

**Table 1:** Basic information of rats

	Control (n = 15)	DM (n = 11)	DM + shN.C (n = 10)	DM + shPHLPP1 (n = 8)
Blood glucose (mmol/L)	5.72 ± 0.36	25.91 ± 3.42*	24.45 ± 4.16*	22.26 ± 3.28*
Bodyweight (g)	473.47 ± 18.36	348.55 ± 59.57*	349.10 ± 33.09*	370.13 ± 51.43*
Heart weight (g)	1.22 ± 0.05	1.37 ± 0.19*	1.37 ± 0.04*	1.36 ± 0.09*
HW/BW (mg/g)	2.57 ± 0.04	3.96 ± 0.34*	3.94 ± 0.32*	3.72 ± 0.37*

Note

Data are expressed as the means ± SD. Statistical analysis was performed using one‐way ANOVA followed by Scheffe's test.

Abbreviations: Control, normal rats; DM, diabetes mellitus; HW/BW, heart weight/bodyweight; shN.C, negative control shRNA; shPHLPP1, PHLPP1 shRNA.

* *P* < .05 compared with controls.

### 3.2 PHLPP1 down‐regulation attenuated cardiac dysfunction in diabetes

**Figure 2:**
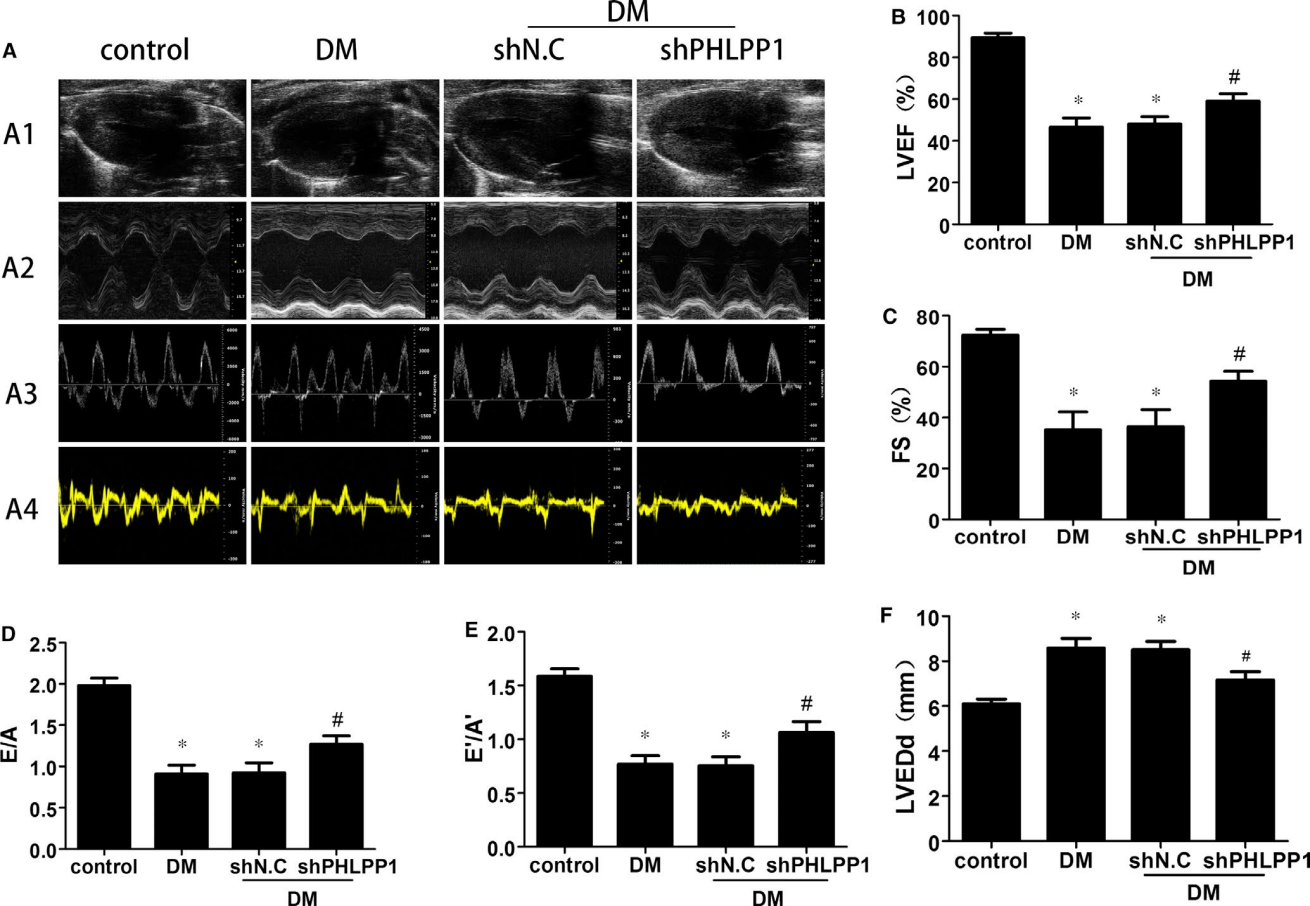
Echocardiographic measurements of control and diabetic rat hearts (n = 8 per group). A1, Representative 2D echocardiograms. A2, Representative M‐mode echocardiograms. A3, Representative pulse‐wave Doppler echocardiograms of mitral inflow. A4, Representative tissue Doppler echocardiograms. B, LV ejection fraction (LVEF). C, Fractional shortening (FS). D, Early‐to‐late mitral flow (E/A). E, Diastolic velocity ratio (E′/A′). F, Left ventricular end‐diastolic dimension (LVEDd). Control: normal rats. DM: diabetes mellitus. shN.C: negative control shRNA. shPHLPP1: PHLPP1 shRNA. All experiments were performed at least 3 times. Data are expressed as the means ± SD. Statistical analysis was performed using one‐way ANOVA followed by Bonferroni's post hoc test. *P < .05 compared with controls; #*P* < .05 compared with DM or shN.C in DM

Sixteen weeks after diabetes induction, echocardiography showed that LVEF, FS, the E/A ratio and the E′/A′ ratio in DM group was significantly decreased than control group and PHLPP1 knock‐down reversed this reduction compared with vehicle group (*P* < .05) (Figure 2A‐E). Moreover, LVEDd was moderate higher in diabetic rats than that in control rats, and PHLPP1 knock‐down attenuated wall thickening compared with vehicle group (*P* < .05) (Figure 2A,F).

### 3.3 PHLPP1 knock‐down alleviated diabetes‐induced myocardial apoptosis and fibrosis

**Figure 3:**
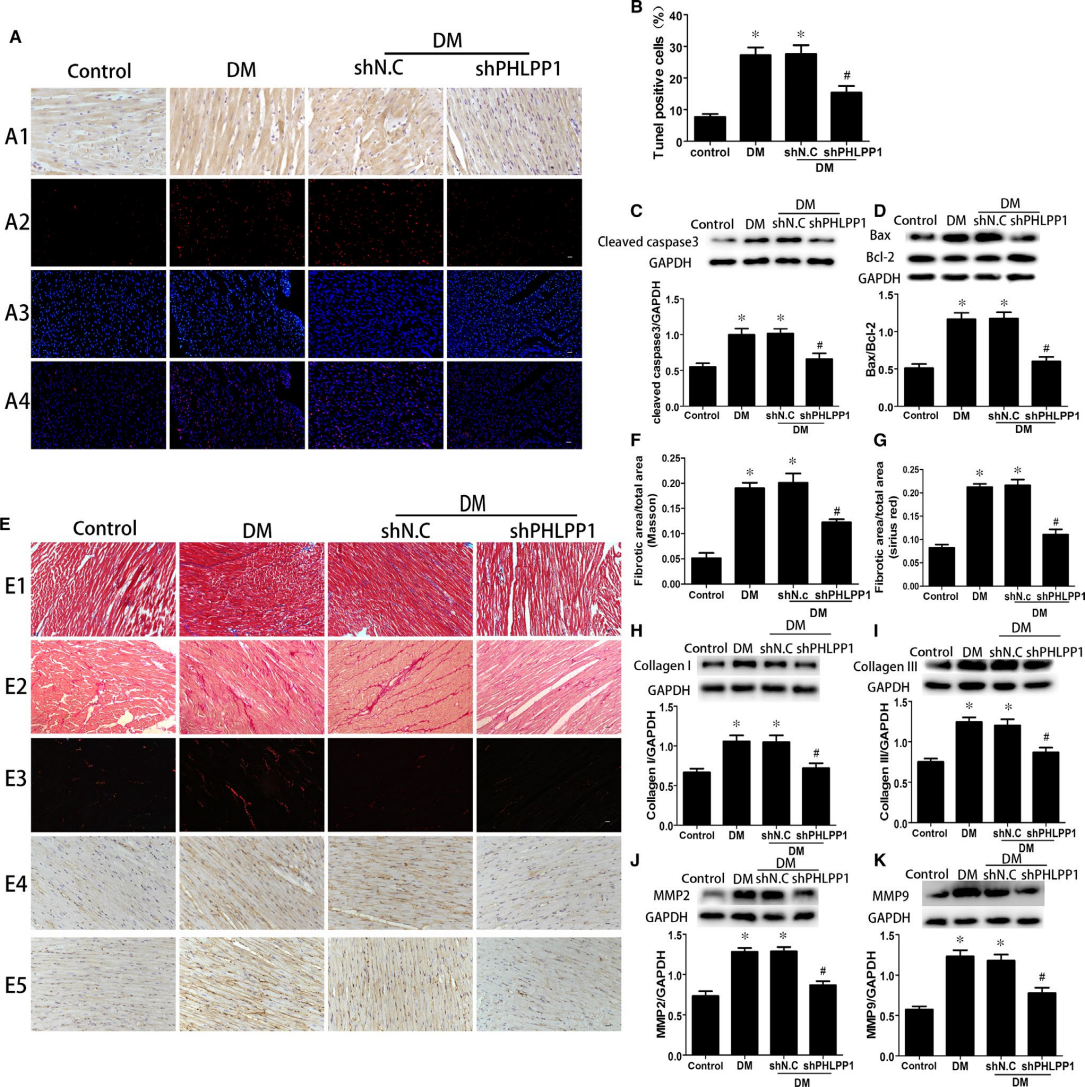
Effects of PHLPP1 on myocardial apoptosis and fibrosis. A, Immunostaining of PHLPP1 (first row, n = 6) and cell apoptosis as determined by TUNEL assay (second‐fourth row, n = 5): apoptosis cell stained red; nuclei stained blue with DAPI. B, Cell apoptosis rate determined by TUNEL assay. C, The levels of cleaved caspase‐3 following PHLPP1 inhibition were measured by Western blot (n = 6). D, The levels of Bax and Bcl‐2 following PHLPP1 inhibition were measured by Western blot (n = 6). E, Representative Masson's trichrome staining (first row) and Sirius red staining (second and third rows) of the myocardium (n = 6). Immunostaining of collagen I (fourth row) and collagen III (fifth row) (n = 6). F, Quantification of Masson's trichrome staining (n = 6). G, Quantification of Sirius red staining (n = 6). H‐K, Western blot analysis of the protein expression of collagen I (F), collagen III (G), MMP2 (H) and MMP9 (I) (n = 6). Control: normal rats. DM: diabetes mellitus. shN.C: negative control shRNA. shPHLPP1: PHLPP1 shRNA. All experiments were performed at least 3 times. Data are expressed as the means ± SD. Statistical analysis was performed using one‐way ANOVA followed by Bonferroni's post hoc test. *P < .05 compared with control, and #*P* < .05 compared with DM or shN.C in DM

Diabetic rat hearts showed remarkably increased TUNEL‐positive apoptotic cells compared with control group (*P* < .05) (Figure 3A,B). Meanwhile, the level of cleaved caspase‐3 as well as Bax/Bcl‐2 ratio was also expressively elevated in diabetic group (*P* < .05) (Figure 3C,D). However, PHLPP1 down‐regulation effectively reduced those increment (*P* < .05) (Figure 3A‐D). Masson's trichrome and Sirius red staining of heart sections demonstrated that collagen deposition in the hearts of diabetic animals was worse than the control animals, and PHLPP1 down‐regulation reduced the collagen deposition compared with vehicle group (*P* < .05) (Figure 3E‐G). Western blot showed that collagens I, collagen III, MMP2 and MMP9 protein levels were elevated in diabetic rat compared with control rats (*P* < .05) (Figure 3H‐K), whereas PHLPP1 down‐regulation reduced the expression of each protein compared with vehicle group (*P* < .05) (Figure 3H‐K).

### 3.4 HG increased the expression of PHLPP1 in primary neonatal rat cardiomyocytes and H9c2 cardiomyoblasts

**Figure 4:**
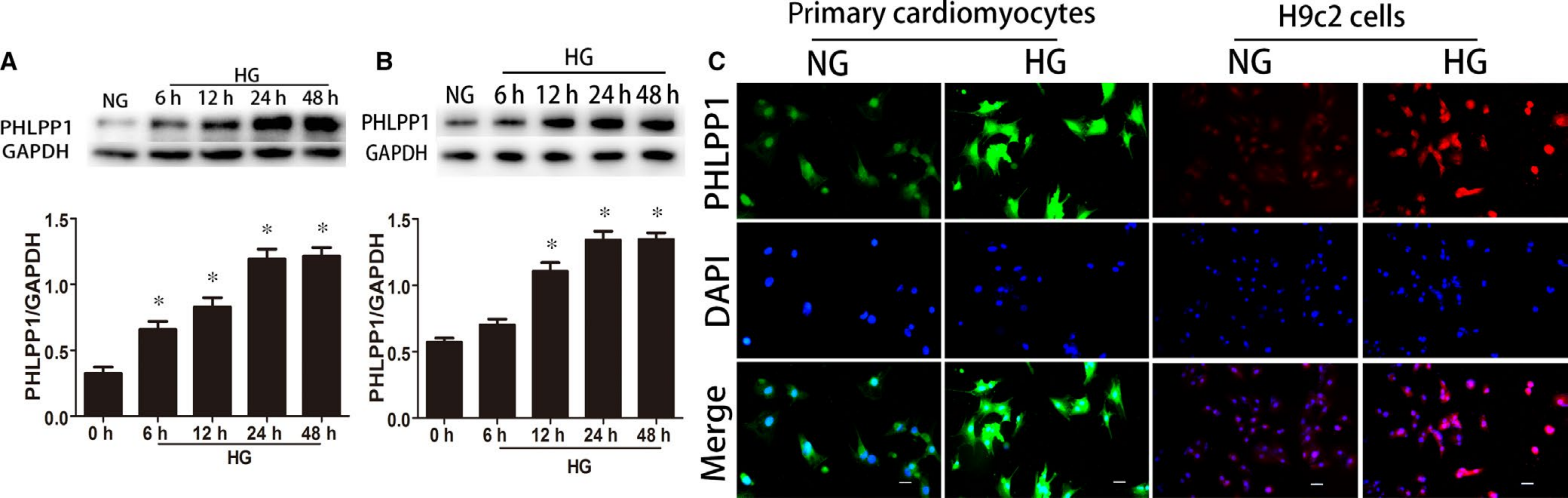
The effects of different glucose culture times on PHLPP1 expression. A, Western blot analysis of the expression of PHLPP1 in primary neonatal cardiomyocytes. B, Western blot analysis of the expression of PHLPP1 in H9c2 cells. C, Immunofluorescence of PHLPP1 in primary neonatal cardiomyocytes and H9c2 cells. PHLPP1 stained green in primary neonatal cardiomyocytes and red in H9c2 cells; nuclei stained blue with DAPI. All experiments were performed at least 3 times.NG: 5.5 mmol/L glucose, HG: 33.3 mmol/L glucose. Data are expressed as the means ± SD. Statistical analysis was performed using one‐way ANOVA followed by Dunnett's multiple‐to‐one comparison test. *P < .05 compared with NG

The PHLPP1 protein level showed a significant increase after 24 hours HG treatment in both primary neonatal cardiomyocytes (*P* < .05) (Figure 4A) and H9c2 cardiomyoblasts (*P* < .05) (Figure 4B). Immunofluorescence microscopy revealed that PHLPP1 is located in the nucleus, the cytoplasm and membrane of both primary cardiomyocytes and H9c2 cardiomyoblasts. Furthermore, culturing in HG environment significantly increased PHLPP1 expression (Figure 4C).

### 3.5 ROS mediated HG‐induced PHLPP1 overexpression

**Figure 5:**
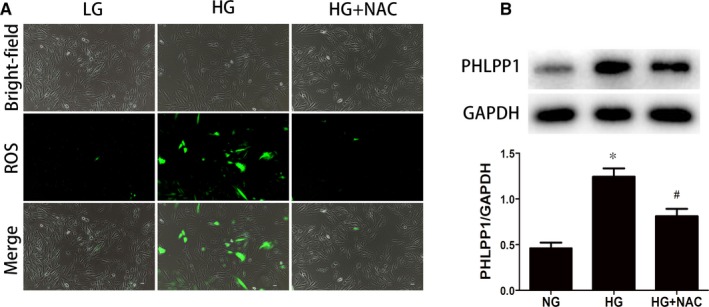
High glucose up‐regulates the expression of PHLPP1 via increasing ROS. A, The production of ROS. 6‐h high‐glucose treatment increased ROS production and this overproduction was inhibited under pretreatment with NAC. B, The protein expression levels of PHLPP1 when pretreated with NAC before 24 h HG‐stimuli. 24 High glucose increased PHLPP1 expression while pretreatment with NAC attenuated this overexpression. NG: 5.5 mmol/L glucose; HG: 33.3 mmol/L glucose; NAC: N‐acetyl cysteine. All experiments were performed at least 3 times. Data are expressed as the means ± SD. Statistical analysis was performed using one‐way ANOVA followed by Bonferroni's post hoc test. *P < .05 compared with NG. #*P* < .05 compared with HG

HG treatment significantly induced ROS overproduction (*P* < .05) (Figure 5A). To explore the role of ROS in the HG‐induced overexpression of PHLPP1 in H9c2 cells, we used a ROS inhibitor, N‐acetyl cysteine (NAC, 3 mmol/L) to inhibit the production of ROS. Immunofluorescence microscopy showed that 6 hours HG stimulation significantly increased ROS production (Figure 5A). What's more, Western blot showed that 24‐hour high‐glucose treatment significantly increased PHLPP1 expression in H9c2 cardiomyoblasts. And pretreatment with NAC (3 mmol/L) attenuated the overexpression of PHLPP1 in the HG‐treated H9c2 cardiomyoblasts (Figure 5B) (*P* < .05).

### 3.6 HG induced H9c2 cardiomyoblasts apoptosis, and PHLPP1 inhibition ameliorated HG‐induced apoptosis of H9c2 cardiomyoblasts

**Figure 6:**
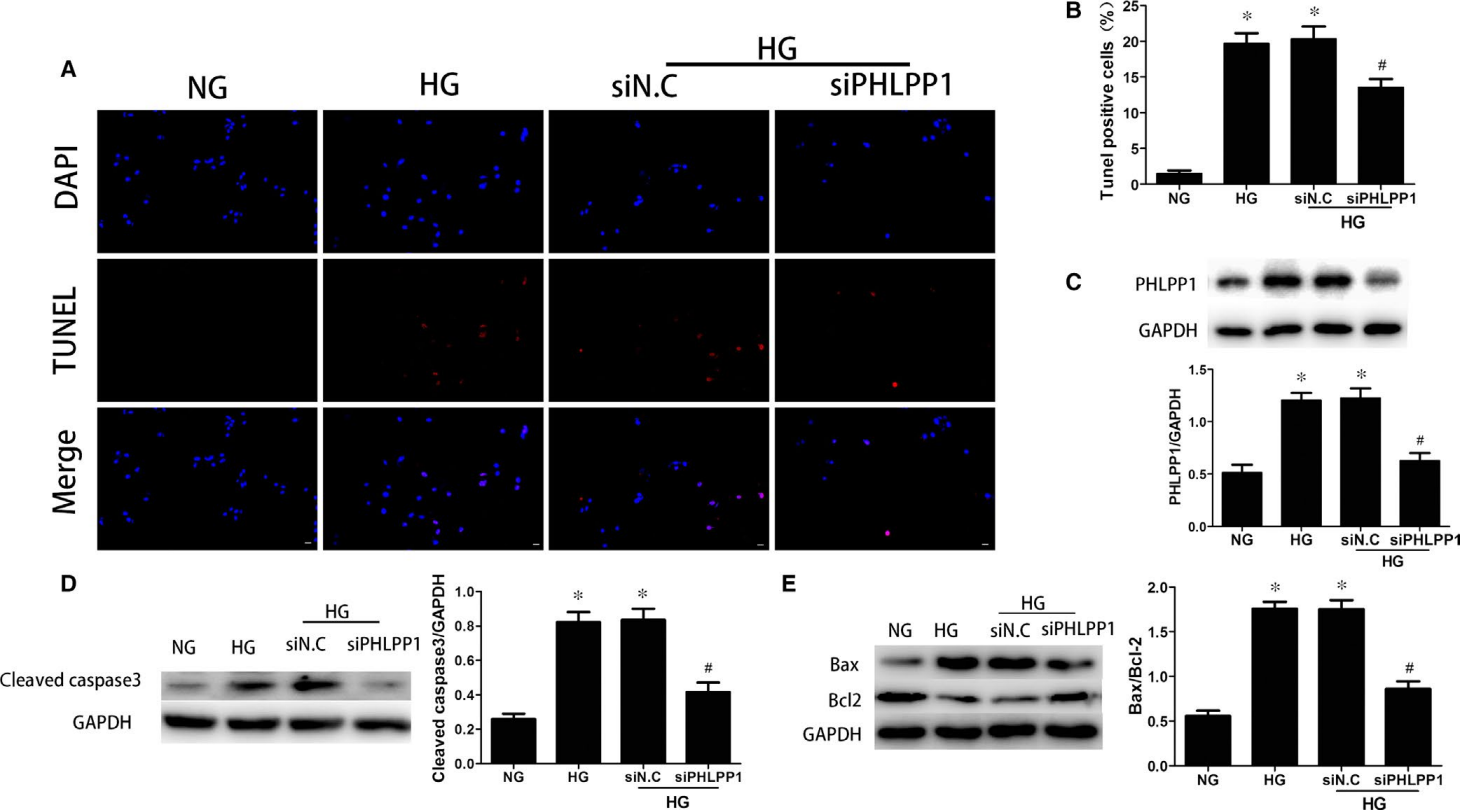
TUNEL analyses and Western blotting demonstrated that PHLPP1 induced apoptosis in H9c2 cardiomyocytes. A‐B, The cell apoptosis rate detected by TUNEL were reduced 24 h after PHLPP1 inhibition. C, The expression of PHLPP1 detected by Western blotting. D‐E, The expression of cleaved caspase‐3 and the Bax/Bcl‐2 ratio was reduced after the expression of PHLPP1 was inhibited. All experiments were performed at least 3 times. NG: 5.5 mmol/L glucose; HG: 33.3 mmol/L glucose. siN.C: negative control siRNA. siPHLPP1: PHLPP1 siRNA. Data are expressed as the means ± SD. Statistical analysis was performed using one‐way ANOVA followed by Bonferroni's post hoc test. *P < .05 compared with NG. #*P* < .05 compared with HG

PHLPP1‐siRNA plasmids were transfected in H9c2 cardiomyoblasts 24 hours prior to HG treatment. After exposure to HG for 24 hours, overexpression of PHLPP1, cleaved caspase‐3 and elevated ratio of Bax to Bcl‐2 (*P* < .05) (Figure 6C‐E) were observed in H9c2 cardiomyoblasts. The TUNEL assay showed that the apoptosis rate was also increased significantly (*P* < .05) (Figure 6A,B). After inhibiting the expression of PHLPP1 with PHLPP1‐siRNA, all of them were significantly down‐regulated (*P* < .05) (Figure 6A‐E). These data demonstrated that HG‐induced apoptosis can be attenuated by the inhibition of PHLPP1.

### 3.7 Inhibition of PHLPP1 reverted HG‐induced p‐PI3K, p‐AKT and p‐mTOR inactivation in the H9c2 cardiomyoblasts

**Figure 7:**
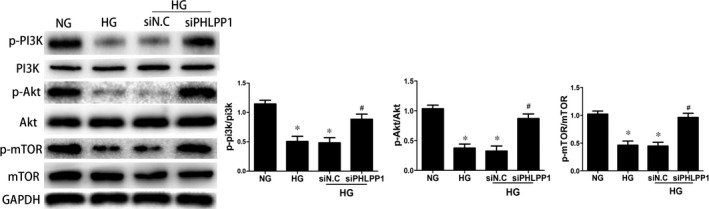
Signal transduction mechanisms involved in the induction of apoptosis by PHLPP1. PHLPP1‐siRNA plasmids were transfected 24 h prior to HG stimulation in H9c2 cell. Western blot analyses of p‐PI3K, p‐Akt and p‐mTOR expression in H9c2 cardiomyoblasts. The expression of p‐PI3K, p‐Akt and p‐mTOR were increased after PHLPP1 inhibition. NG: 5.5 mmol/L glucose; HG: 33.3 mmol/L glucose. siN.C: negative control siRNA. siPHLPP1: PHLPP1 siRNA. All experiments were performed at least 3 times. Data are expressed as the means ± SD. Statistical analysis was performed using one‐way ANOVA followed by Bonferroni's post hoc test. *P < .05 compared with NG. #*P* < .05 compared with HG

PHLPP1‐siRNA plasmids were transfected 24 hours prior to HG stimulation in H9c2 cardiomyoblasts. Western blot analysis suggested that HG treatment significantly reduced PI3K, AKT and mTOR phosphorylation compared with the control group. However, these changes were reversed after inhibition of PHLPP1 (*P* < .05) (Figure 7).

### 3.8 Inhibition of PHLPP1 prevented HG‐induced apoptosis via the PI3K/AKT/mTOR pathway in the H9c2 cardiomyoblasts

**Figure 8:**
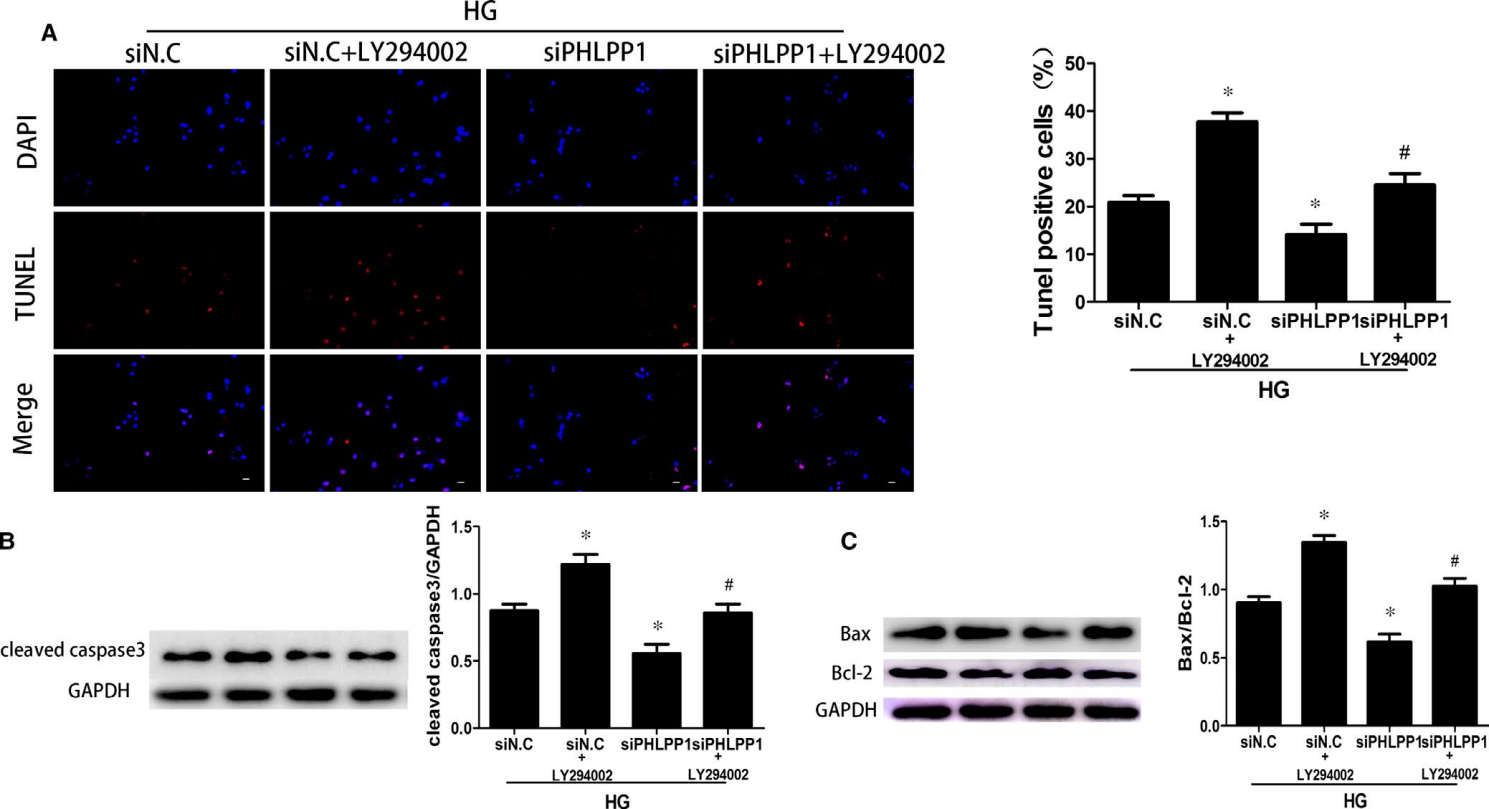
TUNEL analyses and Western blotting demonstrated that LY294002 effectively reversed the anti‐apoptotic effect of PHLPP1 inhibition. A, The cell apoptosis rates detected by TUNEL were reduced after PHLPP1 inhibition; however, this reduction was reversed after pretreated with LY294002. B, The expression of cleaved caspase3 was reduced after PHLPP1 inhibition, while LY294002 reversed this reduction. C, The ratio of Bax to Bcl2 was reduced after PHLPP1 inhibition but it was enhanced when treatment with LY294002. NG: 5.5 mmol/L glucose; HG: 33.3 mmol/L glucose. siN.C: negative control siRNA. siPHLPP1: PHLPP1 siRNA. All experiments were performed at least 3 times. Data are expressed as the means ± SD. Statistical analysis was performed using one‐way ANOVA followed by Bonferroni's post hoc test. *P < .05 compared with HG + siNG. #*P* < .05 compared with HG + siPHLPP1

Twenty‐four hours after down‐regulating PHLPP1 expression with PHLPP1‐siRNA, H9c2 cardiomyoblasts were treated with a PI3K inhibitor, LY294002 (MCE, 25 μmol/L) 2 hours prior to the 24 hours of HG treatment. TUNEL assay showed that the proportion of apoptotic cells was increased in the HG + siN.C + LY294002 group compared with the HG + siN.C group, decreased in the HG + siPHLPP1 group compared with the HG + siN.C group and significantly increased in the HG + siPHLPP1 + LY294002 group compared with the HG + siPHLPP1 group (*P* < .05) (Figure 8A). Consistent with these findings, the expression of cleaved caspase‐3 and the ratio of Bax to Bcl‐2 increased upon LY294002 treatment (*P* < .05) (Figure 8B,C).

## 4 DISCUSSION

Diabetes mellitus has been recognized as a significant health problem worldwide.^26^ Diabetic cardiomyopathy is one of the most important complications of diabetes mellitus, which is distinguished by initial impairment of left ventricular (LV) relaxation followed with LV contractile dysfunction, independent of hypertension, coronary artery disease or other heart diseases.^27^, ^28^, ^29^ The potential pathophysiological mechanisms of diabetic cardiomyopathy including oxidative stress,^8^, ^9^ interstitial fibrosis^10^ as well as myocardial apoptosis,^2^ remain poorly understood.

Pleckstrin homology (PH) domain leucine‐rich repeat protein phosphatase 1 (PHLPP1) is a kind of serine/threonine phosphatase,^13^ whose dysregulation is associated with numerous human diseases. PHLPP1 is known to directly dephosphorylate the hydrophobic motif of Akt (Ser473) to keep the balance between cell survival and apoptosis in several cancers.^30^ Recently, Moc C and colleagues have shown that the deletion of PHLPP1 in cardiomyocytes protects against pathological hypertrophy.^31^ Moreover, Ma and colleagues found that PHLPP1 inhibition potentiated Akt phosphorylation at Ser473 and protected cardiomyocytes from MI/R injury.^32^ Furthermore, some researchers found that the mTORC2/PHLPP1/Akt complex controlled chaperone‐mediated autophagy can regulate cell death.^33^, ^34^ During diabetes, the balance between macromolecules/organelle synthesis and their degradation is hampered. Apoptosis and aberrant autophagy were major causes of cell death and were crucial in the progression of DCM.

In our present study, diabetic rat hearts showed elevated PHLPP1 expression. We found the novel result that the down‐regulating PHLPP1 expression can improve cardiac function and remodelling, alleviate collagen deposition, attenuate HG‐induced cardiomyocytes apoptosis, restore PI3k/Akt/mTOR pathway activity. Thus, PHLPP1 plays a pivotal role in the progression of DCM.

Long‐term hyperglycaemia and hyperglycaemia‐induced oxidative stress are chief reasons for the development of DCM.^8^, ^35^ Additionally, cardiac oxidative stress is associated with cell death and myocardial fibrosis, leading to potential fatal cardiac events.^36^ It occurs under the condition of overproduction of ROS. In our current study, high glucose induced overexpression of PHLPP1 in both primary neonatal rat cardiomyocytes and H9c2 cardiomyoblasts. And high‐glucose treatment results in excessive synthesis of ROS in H9c2 cardiomyoblasts, while inhibition of ROS production decreased PHLPP1 expression. These results indicated that oxidative stress might participate in PHLPP1 overexpression in high glucose cultured H9c2 cardiomyoblasts.

Continuous ventricular cardiomyocytes loss is a unique hallmark of diabetic cardiomyopathy.^2^ PHLPP1 was first discovered as the phosphatases for Akt, which specifically dephosphorylated the hydrophobic motif of Akt at Ser473 site. Thus, PHLPP1 can inactivate Akt to trigger apoptosis and act as a tumour suppressor in multiple cancers.^14^, ^30^ Recently, a study showed that PHLPP1 knock‐down alleviated ischaemic injury in aged heart.^32^ Nevertheless, whether PHLPP1 can play a part in DCM directly has not been characterized. In our present report, TUNEL assay suggested that the apoptosis of cardiomyocytes could be attenuated by PHLPP1 down‐regulation in both HG‐treated H9c2 cardiomyoblasts and diabetic hearts. Caspase 3, as a pivotal executioner of cell apoptosis, gains no activity until it is cleaved by an initiator after the stimulation of apoptotic signalling.^37^ Bax is a BH3 domain‐containing protein that can bind to Bcl‐2 which allows Bax to take an active conformation and enhance apoptosis through attenuating the interaction between Bax and Bcl‐2.^38^ Our study showed that suppressing PHLPP1 expression led to reduced HG‐enhanced caspase‐3 activity and Bax/Bcl‐2 ratio. These results revealed that PHLPP1 down‐regulation could protect cardiomyocytes against HG‐induced apoptosis.

The structural unit of PHLPP1 comprises a C‐terminal PDZ binding motif, a phosphatase domain (PP2C), leucine‐rich repeat region and an N‐terminal domain. The multiple regulatory domains and phosphatase activity allow PHLPP1 to modulate numerous effector molecules.^13^ It has been reported that PHLPP1 could negatively regulate Akt, PKC, Raf, S6K1 and MST1 activity, thereby affecting cell survival and cell death.^18^, ^19^ PI3K is a crucial orchestrator of glucose regulation, suggesting its possible involvement in the onset of diabetes mellitus.^24^ PI3K generates the lipid second messenger phosphatidylinositol‐3,4,5‐trisphophate (PIP3) which provides a docking site for the PH domain of Akt, resulting in phosphorylation of Akt.^39^ Akt is of great importance in regulating the balance between cell survival and apoptosis. Phosphorylation of Akt at the hydrophobic motif and the activation loop activates the kinase and promotes cell survival.^40^ Finally, Akt activates mTORC1 by phosphorylation of tuberous sclerosis complex 2 (TSC2) to regulate cell survival.^22^ Previous studies showed that PHLPP1 could directly dephosphorylate the hydrophobic motif of Akt (Ser473) to promote tumour cell survival.^40^ However, the majority of the studies on PHLPP1 have been conducted in cancer cells, with little information regarding the function of PHLPP1 in the heart, especially in diabetic heart. To clarify whether attenuation of cardiomyocytes apoptosis resulted from PHLPP1 inhibition is mediated by activating PI3K/Akt/mTOR pathway, the level of P‐PI3K, PI3K, P‐Akt, Akt, p‐mTOR and mTOR was analysed. In our study, HG incubation appeared to selectively inhibit PI3K/Akt/mTOR pathway, while PHLPP1 inhibition increased the activity of this pathway.

To further confirm that down‐regulation of PHLPP1 prevent HG‐induced cardiomyocytes apoptosis via activating PI3K/AKT/mTOR pathway, we pretreated H9c2 cardiomyoblasts with LY294002, 2 hours before HG stimulation. Both TUNEL assay and Western blotting indicating that LY294002 effectively reversed the anti‐apoptotic effect of PHLPP1 inhibition. Taken together, these data demonstrated that the protective effects of PHLPP1 inhibition were abolished by LY294002, the PI3K/Akt/mTOR pathway inhibitor. Inhibition of PHLPP1 prevents HG‐induced apoptosis via activating the PI3K/AKT/mTOR pathway.

In addition to cardiomyocyte apoptosis, intensive cardiac fibrosis is another crucial characteristic of DCM.^41^, ^42^ Excess production and deposition of ECM, principally collagen I and III, can alter the structure of the heart and lead to severe cardiac dysfunction.^10^, ^42^ And HG stimulates MMP‐2 and MMP‐9 expression in diabetic hearts which accelerates tissue remodelling and cardiac fibrosis. Foetal gene re‐expression and natriuretic peptides up‐regulation indicated reduction of cardiac function decreased. In our study, shPHLPP1 treatment reduced β‐MHC re‐expression and BNP up‐regulation in diabetic ret hearts. And diabetic‐induced aberrant deposition of collagen I and III as well as increased expression of MMPs were reduced after shPHLPP1 treatment. However, we have not explored the mechanisms of PHLPP1 inhibition reduced cardiac fibrosis.

The data presented here demonstrate that PHLPP1 gene silencing improves myocardial function and protects DCM from myocardial apoptosis and fibrosis. Moreover, PHLPP1 down‐regulation plays a crucial role in hyperglycaemia‐induced cardiomyocyte apoptosis via activating the PI3K/AKT/mTOR pathways. However, the effects and molecular mechanism of PHLPP1 inhibition in myocardial fibroblasts have not been fully elucidated. Further investigations using myocardial fibroblasts and spontaneous diabetes model, such as db/db mice or gene knockout mice should be needed to uncover these mechanisms. Given the cardio‐protective effects of PHLPP1 inhibition, PHLPP1 may be a novel therapeutic target in the treatment of DCM.

## 5 CONCLUSIONS

In summary, PHLPP1 plays an important part in the progression of diabetic cardiomyopathy. Oxidative stress participates in high glucose‐induced PHLPP1 overexpression in H9c2 cardiomyoblasts. PHLPP1 inhibition can directly relieve cardiac remodelling and ameliorates cardiac dysfunction via inhibiting apoptosis and fibrosis in DCM. Moreover, PHLPP1 inhibition can reduce HG‐induced H9c2 cardiomyoblasts apoptosis through increasing the phosphorylation of the PI3K/Akt/mTOR signalling pathway.

## CONFLICT OF INTEREST

The authors declare that there is no conflict of interest.

## AUTHORS' CONTRIBUTIONS

M. Zhang and X. Wang were responsible to induce animal model and cell experiment. J. Tian and M. Liu performed immunohistochemistry staining and other staining. Y. Xu and D. Liu analysed and interpreted the animal model data. T. Jin and J. Pan performed the ultrasonic cardiogram examination of heart. F. An was a major contributor in writing the manuscript and Western blot experiment. All authors read and approved the final manuscript.

## Data Availability

All data generated or analysed during this study are included in this article and its additional information files.
